# *Rickettsia* ‘In’ and ‘Out’: Two Different Localization Patterns of a Bacterial Symbiont in the Same Insect Species

**DOI:** 10.1371/journal.pone.0021096

**Published:** 2011-06-21

**Authors:** Ayelet Caspi-Fluger, Moshe Inbar, Netta Mozes-Daube, Laurence Mouton, Martha S. Hunter, Einat Zchori-Fein

**Affiliations:** 1 Department of Entomology, Newe-Ya'ar Research Center, ARO, Ramat-Yishay, Israel; 2 Department of Evolutionary and Environmental Biology, University of Haifa, Haifa, Israel; 3 Laboratoire de Biométrie et Biologie Evolutive (UMR-CNRS 5558), Université Claude Bernard—Lyon1, Villeurbanne, France; 4 Department of Entomology, University of Arizona, Tucson, Arizona, United States of America; University of Poitiers, France

## Abstract

Intracellular symbionts of arthropods have diverse influences on their hosts, and their functions generally appear to be associated with their localization within the host. The effect of localization pattern on the role of a particular symbiont cannot normally be tested since the localization pattern within hosts is generally invariant. However, in Israel, the secondary symbiont *Rickettsia* is unusual in that it presents two distinct localization patterns throughout development and adulthood in its whitefly host, *Bemisia tabaci* (B biotype). In the “scattered” pattern, *Rickettsia* is localized throughout the whitefly hemocoel, excluding the bacteriocytes, where the obligate symbiont *Portiera aleyrodidarum* and some other secondary symbionts are housed. In the “confined” pattern, *Rickettsia* is restricted to the bacteriocytes. We examined the effects of these patterns on *Rickettsia* densities, association with other symbionts (*Portiera* and *Hamiltonella defensa* inside the bacteriocytes) and on the potential for horizontal transmission to the parasitoid wasp, *Eretmocerus mundus,* while the wasp larvae are developing within the whitefly nymph. Sequences of four *Rickettsia* genes were found to be identical for both localization patterns, suggesting that they are closely related strains. However, real-time PCR analysis showed very different dynamics for the two localization types. On the first day post-adult emergence, *Rickettsia* densities were 21 times higher in the “confined” pattern vs. “scattered” pattern whiteflies. During adulthood, *Rickettsia* increased in density in the “scattered” pattern whiteflies until it reached the “confined” pattern *Rickettsia* density on day 21. No correlation between *Rickettsia* densities and *Hamiltonella* or *Portiera* densities were found for either localization pattern. Using FISH technique, we found *Rickettsia* in the gut of the parasitoid wasps only when they developed on whiteflies with the “scattered” pattern. The results suggest that the localization pattern of a symbiont may influence its dynamics within the host.

## Introduction

Intracellular bacterial symbionts are common among terrestrial and marine multicellular organisms and can be found in plants and animals, vertebrates and invertebrates [1]. In arthropods, obligate “primary” symbionts such as *Buchnera* in aphids and *Carsonella* in psyllids [2] have mutualist relationships with their hosts, and provide essential nutrients under limited or unbalanced diets. Primary symbionts are generally localized in specialized cells called bacteriocytes, grouped together in a bacteriome. The bacteriocytes provide the symbionts with a protected environment and is involved in the exchange of amino acids between the host and bacteria [3]. Primary symbionts generally have the ability to penetrate host germ cells and be maternally (vertically) transmitted [2].

Symbionts that are usually not required for the host's survival or reproduction—“secondary symbionts”—nonetheless have important effects on host biology and ecology. Secondary symbionts may manipulate host reproduction in ways that enhance their vertical transmission, or help in the host's defense against thermal stress, natural enemies and pathogens [4]. The localization patterns of secondary symbionts in their hosts are diverse: for example, symbiotic bacteria have been reported in insect tissues such as the Malpighian tubules [5], hemolymph [6], [7], brain [8] and salivary glands [9]. Symbionts that influence the reproduction of their hosts, such as *Wolbachia* and *Cardinium*, are frequently found in the gonads [10]–[13], but *Wolbachia* has also been described in hemocytes [7]. Like primary symbionts, intracellular secondary symbionts are generally vertically transmitted, and are therefore present in the gonads of their hosts regardless of whether they influence host reproduction.

Many arthropod individuals host more than one symbiont, and the bacterial community is thought to have diverse interactions, especially when co-localized in particular tissues. Competition between symbionts, expressed as reduced densities of one in the presence of another, has been hypothesized to occur when the resources provided by the host are limited, for example, when the density of the pea aphid *Acyrtosiphon pisum*'s primary symbiont *Buchnera aphidicola* is depressed in the presence of *Serratia symbiotica* [14] or *Rickettsia* [15]. In contrast, positive interactions between strains of cytoplasmic incompatibility-inducing *Wolbachia* have been reported: the density of each *Wolbachia* strain was higher in the presence of others than it was in a single infection, and contributed to maximum infection in the host [16], [17].

The sweet potato whitefly, *Bemisia tabaci* (Gennadius) (Homoptera: Aleyrodidae), harbors a primary symbiont, *Portiera aleyrodidarum*, which is restricted to the bacteriocytes and produces amino acids lacking in the phloem diet [2]. In addition, *B. tabaci* can host a variety of secondary symbionts with unknown function: *Arsenophonus*, *Cardinium, Fritschea, Wolbachia, Hamiltonella*, and *Rickettsia* [2], [18], [19].

Gottlieb et al. [20] described two different localization patterns of *Rickettsia* in *B. tabaci*. In the “scattered” (S) localization pattern, *Rickettsia* is distributed throughout the whitefly's body, excluding bacteriocytes, in all of the developmental stages of *B. tabaci* except newly laid eggs (in young eggs, the bacterium is found only in the bacteriocytes). In the “confined” (C) localization pattern, *Rickettsia* is restricted to the bacteriocytes at all developmental stages tested. Interestingly, Chiel et al. [21] have shown that S-pattern *Rickettsia* can be transmitted to parasitoid wasps that develop within the whitefly nymph. The parasitioid wasp *Eretmocerus* sp. nr. *emiratus* is infected throughout adulthood with the bacterium, but *Rickettsia* is not transmitted to the next generation.

We took advantage of this unique localization diversity by establishing *B. tabaci* lines with the S and C *Rickettsia-*localization patterns to address the following questions:

1. Are the two localization patterns produced by genetically distinct *Rickettsia* strains?
2. Does localization pattern affect the density of *Rickettsia*?
3. Is there a correlation between *Rickettsia* localization pattern and the density of *Portiera* and *Hamiltonella* inside the bacteriocytes?
4. Can *Rickettsia* with both C and S localization be horizontally transmitted to parasitoid wasps?

## Materials and Methods

### Insects

#### Whitefly rearing and strains

Whiteflies from three different *Bemisia tabaci* B biotype colonies were used: lines SSC and R^+^ which harbor *Rickettsia* with S and C localization patterns respectively, and a *Rickettsia-*free (R^-^) line (Table 1). The lines, received from Prof. Gerling and Dr. Ghanim on 2006, were reared on cotton (*Gossypium hirsutum* ‘Acala’) under standard greenhouse conditions: 26±2°C, 60% RH (relative humidity), and a photoperiod of 14∶10 h (light/dark) in Newe Ya'ar.

**Table 1:** *Bemisia tabaci* lines studied.

Line	Symbiont composition^a^	Origin
scattered *Rickettsia* (S)	*Portiera, Rickettsia, Hamiltonella*	SSC strain. Zora, Israel, 1987 (ARO)
confined *Rickettsia* (C)	*Portiera, Rickettsia, Hamiltonella*	Tel Aviv Univ. (R^+^ and R^-^ strains described in chiel et al. [39]).
*Rickettsia*-free (R^-^)	*Portiera, Hamiltonella*	

a Data from Chiel et al. [18], Gottlieb et al. [20].

#### Parasitoid rearing

The parasitoid wasp *Eretmocerus mundus* Mercet (Hymenoptera: Aphelinidae) was reared on broccoli (*Brassica oleracea* var. *botrytis*) plants infested with *B. tabaci* nymphs from one of the three whitefly colonies in separate cages. All three parasitoid cultures were kept under standard greenhouse conditions (26±2°C, 60% RH and 14∶10 h light/dark photoperiod).

#### Gene-sequence comparison between localization patterns

To determine whether the differences between *Rickettsia* S and C localization stem from different strains of the bacterium, four *Rickettsia* genes (*RickA, GroEL, gltA* and *16S rRNA*) were sequenced from each localization pattern. The *16S rRNA* and *gltA* were chosen because they are conserved genes that are commonly used for bacterial classification in general and for that of *Rickettsia* in particular [22]. *RickA* is a *Rickettsia*-specific gene involved in actin tail formation, the machinery that enables bacterial movement within and between cells [23]. Because it was initially hypothesized that differences between localization patterns might stem from *Rickettsia* immobility in the C pattern due to a defect in *RickA*, that gene was fully sequenced. Adults of *B. tabaci* females were placed alive in 96% alcohol and three individuals from each population were ground separately in lysis buffer as described by Frohlich et al. [24]. Fragments of the four genes were amplified using PCR from the insect lysate with specific primer combinations (Table 2). Reactions were performed in a 25-µl volume containing 3 µl of the template DNA lysate, 10 pmol of each primer, 0.2 mM dNTPs, 1X Red Taq buffer and one unit of Red Taq DNA polymerase (Sigma). PCR products were stained with SafeView™ (NBS Biologicals) and visualized on a 1.2% agarose gel. Because *RickA* could not be visualized after one amplification cycle, nested PCR was used to increase the sensitivity of the reaction using a similar PCR routine. After amplification, the product was diluted 1∶100 in water and 3 µl of the dilution was used for another PCR with internal primers (Table 2). Negative controls of the first PCR round were used for the second PCR round after dilution.

**Table 2:** PCR primer sets used in this study.

Gene	Primer set	Nucleotide sequence (5′→3′)	Expected size (bp)	Reference
*Rickettsia 16S rRNA*	Rb-F1513-R	GCTCAGAACGAACGCTATCACGGYTACCTTGTTACGACTT	∼1500	[25]
*Rickettsia gltA*	409-F1273-R	CCTATGGCTATTATGCTTGC CATAACCAGTGTAAAGCTG	∼850	[40]
*Rickettsia RickA*	Trp4-FBcr-RRicka5-F^a^Ricka3-R^a^	GGATTATCTCTCTCATATTTG TCTGCTGCTGCGTTTTATTAT ATGGCAAAGATAACTGAGCT CTACCTTTGTTGAGATTGTT	∼1500	This paper. Designed based on *Rickettsia bellii* genome (NCBI accession NC_007940)
*Rickettsia GroEL*	RGEL-508-FRGEL-stop-R	GGCAAAGAAGGCGTAATAACTG TTAGAAGTCCATACCTCCCA	∼1100	[41]
*Portiera 16S rRNA* (real-time quantitative PCR)	Port73-FPort266-R	GTGGGGAATAACGTACGG CTCAGTCCCAGTGTGGCTG	∼195	This paper. Designed based on *Portiera aleyrodidarum* genome
*Actin B. tabaci* (real-time quantitative PCR)	Wf-B actin-FWf-B actin-R	TCTTCCAGCCATCCTTCTTG CGGTGATTTCCTTCTGCATT	∼200	[42]
*Hamiltonella dnaK* (real-time quantitative PCR)	dnaK-FdnaK-R	GGTTCAGAAAAAAGTGGCAG CGAGCGAAAGAGGAGTGAC	∼200	[43]
*Rickettsia gltA* (real-time quantitative PCR)	glt375-Fglt574-R	TGGTATTGCATCGCTTTGGG TTTCTTTAAGCACTGCAGCACG	∼200	This paper. Designed based on *Rickettsia bellii* genome (NCBI accession NC_007940)

a Internal primers.

PCR products were cloned into the pGEM T-Easy plasmid vector (Promega) and transformed into *Escherichia coli*, and two colonies from each plate were randomly picked and sequenced. For each gene, sequencing was performed, and data obtained from all six replicates (3 individuals ×2 colonies) were used to create consensus sequences. These sequences were compared one to the other and to known sequences in databases using the BLAST algorithm in NCBI.

### *Rickettsia* multiplication rate and interactions with other symbionts

To study the effects of the different localization patterns on *Rickettsia* dynamics and interactions with other symbionts in the host, *Rickettsia, Portiera* and *Hamiltonella* densities were assessed using real-time quantitative PCR. Cotton leaves with *B. tabaci* pupae from the S and C lines were removed from the rearing colony and placed in cages with clean cotton plants. About 50 emerging adults were collected directly into 96% ethanol on days 1 and 21 after emergence.

Amplification of *Rickettsia gltA*, *Hamiltonella dnaK* and *Portiera 16S rRNA* from 1- and 21-day-old adult female whiteflies from both *Rickettsia*-localization lines was performed using 1X Absolute™ QPCR SYBR Green ROX mix (Thermo Scientific) and 5 pmol of each primer (Table 2). *B. tabaci actin* DNA was used as an internal standard for data normalization and quantification [25]. To validate the data, each gene was amplified in duplicate in each of 20 biologically independent replicates. The cycling conditions were: 15 min activation at 95°C, 40 cycles of 15 s at 95°C, 1 min at 60°C. Standard curves were drawn using standard plasmid samples for each symbiont's gene at concentrations of 10^2^, 10^3^, 10^4^, 10^5^ and 10^6^ copies/µl. An ABI Prism® 7000 Sequence Detection System (Applied Biosystems) and accompanying software were used to quantify the real-time quantitative PCR data. The ratios, corresponding to bacterial gene copy number divided by the host nuclear *actin* gene copy number (relative density), were analyzed using R statistical software (http://www.R-project.org). Because of non-normal distribution of the data (Shapiro test), nonparametric tests were used: Kruskal-Wallis for multiple comparisons and Mann-Whitney for 2×2 comparisons. The correlations between symbiont densities were tested using Pearson's product-moment correlation coefficient after log transformation.

### Visualization of *Rickettsia* in the parasitoid wasps

To identify and localize *Rickettsia* in the parasitoid wasp larvae within *B. tabaci*, fluorescence in situ hybridization (FISH) technique was applied. Parasitized *B. tabaci* from each *Rickettsia*-localization pattern (S and C) line were placed in Carnoy's fixative [15]. FISH was performed with symbiont-specific *16S rRNA* for *Rickettsia* and *Portiera* as described by Gottlieb et al. [25]. Stained samples were mounted whole and viewed under a 1X-81 Olympus FluoView 500 confocal microscope. Specificity of detection was confirmed using no-probe staining, RNase-digested specimen staining and *Rickettsia*-free whiteflies.

## Results

### Gene-sequence comparison between localization patterns

To determine whether the two *Rickettsia*-localization patterns found in *B. tabaci* resulted from genetically distinct *Rickettsia* strains, the genes *RickA*, *GroEL*, *gltA* and *16S rRNA* were sequenced. All four sequences, approx. 5000 bp of the *Rickettsia* genome in total, showed 100% identity between the two patterns.

### Influence of host age on *Rickettsia* densities in C and S lines

**Figure 1:**
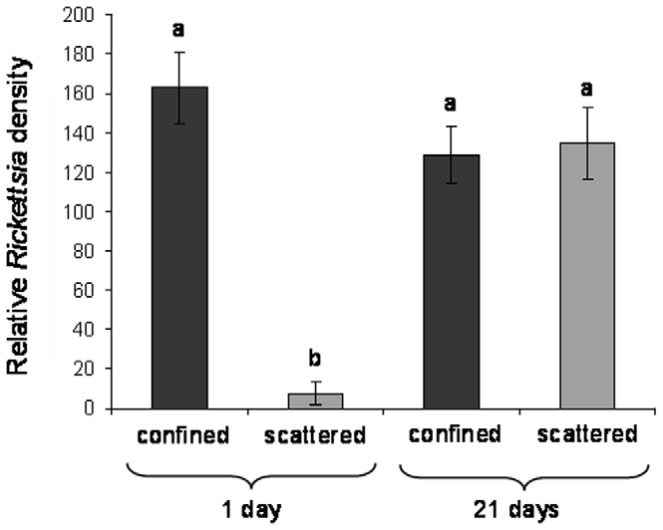
Mean relative *Rickettsia* densities (±SE) (number of copies of the symbiont gene divided by number of copies of the host gene) were evaluated in terms of *gltA* copy number per number of *Bemisia actin* gene copies. Values correspond to the average of 13 to 20 individuals per line. Bars marked with the same letters are not significantly different (Mann-Whitney test, p = 0.05).

*Rickettsia* densities in both localization patterns were compared between young and old adult female whiteflies. For the S pattern, a significant difference was recorded between 1 and 21 days (Mann-Whitney test, U = 5, p<<0.01) (Figure 1). On average, the ratio was 17.5 times higher at 21 days than at 1 day. In contrast, no significant difference was found between 1- and 21-day-old adults from the C line (Mann-Whitney test, U = 254, p = 0.15) (Figure 1). When S and C were compared on day 1, relative density of C-localized *Rickettsia* was 21-fold that of S-localized *Rickettsia*. This difference was significant (Mann-Whitney test, U = 3, p<<0.01). By 21 days, however, the densities of *Rickettsia* in both localization patterns were similar (Mann-Whitney test, U = 175, p = 0.68) (Figure 1).

### Influence of host age in C and S lines on interactions with *Hamiltonella* and *Portiera*

The relative density of *Hamiltonella* was similar for both 1- and 21-day-old adults of both lines (Table 3), (comparisons between all modalities, Kruskal-Wallis, *X* ^2^ = 4.3893, df = 3, p = 0.22; 2×2 comparisons, NS). Similarly, neither whitefly age nor *Rickettsia* localization pattern affected *Portiera* density (Mann-Whitney test, U = 90, p > 0.09).

**Table 3:** Means (± SE) of the bacterial/host gene copy ratio and statistical tests^a^.

Population	Age	*n*	*Portiera*	*Hamiltonella*	*Rickettsia*
**Confined**	**1 day**	14	19.9±6.4 (a)	4.6±0.6	163±18.1 (a)
	**21 days**	15	33.6±6.8 (a,b)	4.0±0.7	128±14.5 (a)
**Scattered**	**1 day**	9	29.4±8.5 (a,b)	3.8±0.4	7.7±6.0 (b)
	**21 days**	13	29.7±2.0 (b)	3.2±0.4	135±18.1 (a)
Kruskal-Wallis test			**0.026**	0.222	**<0.0001**

a Values correspond to the mean number of symbiont gene copies ± SE (*16S rDNA* for *Portiera*, *dnaK* for *Hamiltonella* and *gltA* for *Rickettsia*) divided by the number of nuclear gene copies (*actin* gene). Kruskal-Wallis and Mann-Whitney nonparametric tests were performed with a probability level of significance of 0.05. Means marked with the same letter are not significantly different (Mann-Whitney test). For Kruskal-Wallis tests, p-values are indicated. Bold typeface indicates significant effects. *n* -number of individuals used for the quantifications.

The density of *Rickettsia* was not correlated with the densities of any of the other symbionts tested (Pearson's correlation, r = −0.3097, p = 0.7581 for *Portiera*, r = −0.2537, p = 0.8055 for *Hamiltonella*), but the amounts of *Portiera* and *Hamiltonella* were positively correlated (Pearson's correlation, r = 0.7, p<<0.01). Our data thus show that an individual that harbors relatively more *Hamiltonella* also harbors more *Portiera* (data not shown in the table).

### Horizontal transmission of C- and S-localized *Rickettsia* to parasitoid wasps

**Figure 2:**
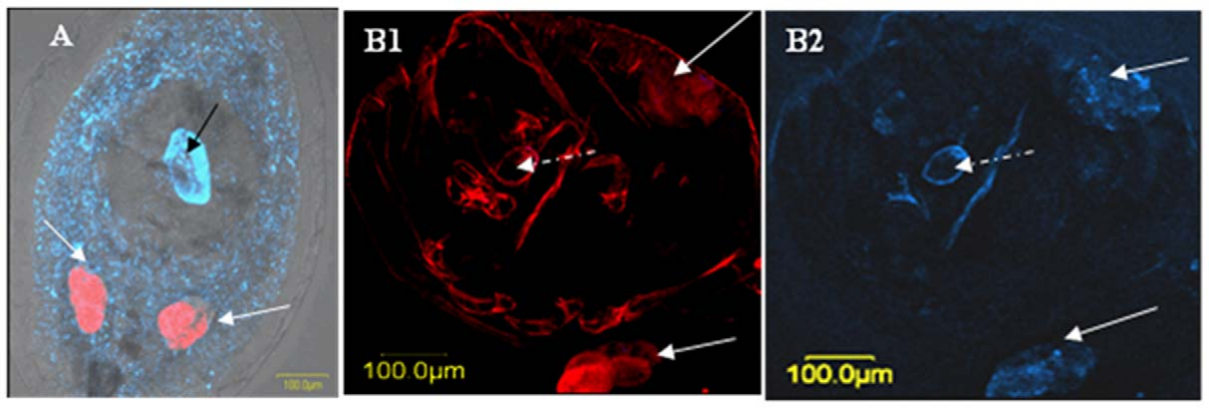
FISH of *Bemisia tabaci* nymphs parasitized by *Eretmocerus mundus*. The procedure was performed using *Portiera*-specific probe (red) and *Rickettsia-*specific probe (blue). (**A**) Scattered (S) localization pattern. White arrows indicate bacteriocytes. Blue-speckled area is the whitefly hemocoel, and the dark, clear area corresponds to the outline of the roughly spherical *Eretmocerus* larva. Bright blue area (black arrow) shows the wasp larval gut. (**B**) Confined (C) localization pattern. (**B1**) Red area (white arrows) shows *Portiera* in the bacteriocytes (**B2**) Blue area (white arrows) shows *Rickettsia* in the bacteriocytes. The pictures of *Rickettsia* and *Portiera* are presented separately because of the faint signal seen by the former. Other blue and red areas in the pictures are due to autofluorescence of the whitefly nymph and the shell of the wasp's hatched egg (white dashed arrow).

FISH technique was used to examine whether the localization pattern of *Rickettsia* influences the potential for its horizontal transmission from whiteflies to their parasitoid wasps. We detected a high concentration of *Rickettsia* in the center of the roughly spherical *E. mundus* larvae developing on S-localization line nymphs. The *Rickettsia* was seen in what appeared to be the parasitoid larva's blind digestive tract (Figure 2A). In contrast, the symbiont could not be detected in wasp larvae developing on C-line whitefly nymphs (Figure 2B).

To verify that *Rickettsia* was not consumed by the larva at a later stage of its development, PCR for *Rickettsia 16S rRNA* was performed on adult wasps that developed on C whiteflies. The results confirmed the lack of detection of *Rickettsia* in any of the wasps that developed on C whitefly nymphs (Elad Chiel, pers. comm.).

## Discussion

We found two localization patterns of a secondary symbiont, *Rickettsia* in different individuals of the same whitefly host. This variation in *Rickettsia* localization may be the result of a genetic modification in host factors that control symbiont movement or of a change in the bacterium that affects its mobility. Changes in bacterial movement may be further influenced by environmental conditions (temperature, humidity, etc.). We found the four *Rickettsia* genes in the whitefly S and C lines to be 100% identical, suggesting that these two bacteria are very closely related, if not identical. Although these findings support the hypothesis that host genes are involved in this peculiar phenotype, further genomic characterization could reveal that differences in more variable regions, or even a single base pair change in the bacterium, are causing it.

Adult whiteflies with the S localization pattern emerged with relatively low densities of *Rickettsia* compared to those with the C pattern. We do not know if this difference corresponds to lower densities in the S whitefly nymphs, or if the titer of *Rickettsia* changes during incomplete metamorphosis to the adult stage. Regardless, as the whitefly ages, the density of the *Rickettsia* in the S line increases until it equals the amount found in the C line at both 1 and 21 days, suggesting that the bacterium is only multiplying in the S adults (Figure 1). The density of *Rickettsia* found in C whiteflies was 21 times higher on day 1 than that found in the S line. Adult females live up to 4 weeks [26] and lay most of their eggs in the first few weeks [26], [27], and therefore a 21-day-old female is close to the end of her productive life. The current setup of our experiments does not allow us to determine whether the observed proliferation of *Rickettsia* occurs gradually throughout the adult's life, or is simply a result of the host's reduced ability to control symbiont quantities with age. However, the fact that both localization patterns reach the same *Rickettsia* density is very interesting and may suggest a threshold regulated by either the bacteria or the host.

We tested the hypothesis that competition between *Rickettsia* with the C localization pattern and other symbionts in the bacteriocytes (*Portiera* and *Hamiltonella*) will be greater than that between these bacteriocyte residents and the *Rickettsia* with the S localization pattern, due to the physical separation of the latter. The results did not support this hypothesis and instead showed no influence of *Rickettsia* on the numbers of either *Portiera* or *Hamiltonella*, irrespective of localization pattern. Instead, the analysis suggests that the titer of each of the symbionts measured is regulated independently. In contrast to our results, in the pea aphid *Acyrthosiphon pisum*, *Rickettsia* suppressed the density of the primary symbiont *Buchnera* when they were co-localized in the bacteriocytes [15]. On the other hand, the number of *Hamiltonella* was found to be positively correlated with that of *Portiera* in both C and S whiteflies, meaning that an individual that harbors more *Hamiltonella* is also likely to harbor more *Portiera.*

The length of the evolutionary relationship between symbiont and host can also influence localization pattern. A newly acquired symbiont sometimes has deleterious effects on its host, which may be reduced over longer selection periods [28]. Thus, symbionts that are closely associated with their hosts are more likely to be localized in bacteriocytes and in the fat body, where they might synthesize essential metabolites. Those symbionts might play an important role in their host's life and are maternally transmitted. Symbionts in the hemolymph, salivary glands and secreting organs might be less likely to contribute to their host's diet, and are more likely to be horizontally transmitted. For example, *Rickettsia felis,* a pathogen of warm-blooded animals that has been identified in the salivary glands of the cat flea, *Ctenocephalides felis*, has been shown to be horizontally (as well as vertically) transmitted [8].

Here we show that horizontal transmission of *Rickettsia* to the parasitoid wasp *E. mundus* is influenced by its localization in the whitefly host. *Rickettsia* could be visualized in the parasitoid wasp larvae only when the larva was reared on S nymphs. S-localized *Rickettsia* is consumed throughout larval development as the larva begins development in its living host by ingesting whitefly hemolymph and tissues. Eventually, the wasp larva kills the whitefly and consumes all remaining tissue. *Rickettsia* in the C localization pattern, which was restricted to the bacteriocytes, was not seen in the larval digestive track of the wasp, and PCR of adult wasps confirmed that they had not consumed the *Rickettsia* from the whitefly host. Although the influence of *Rickettsia* on both the whitefly and its parasitoid is still not clear, the fact that the wasp is exposed to the bacterium in one host but not the other may have a considerable effect on diverse aspects.

*Rickettsia* in whiteflies is not the only example of a symbiont with more than one localization pattern. In pea aphids, *Rickettsia*, *Hamiltonella*, *Serratia* and *Regiella* are found in three locations within their hosts: secondary bacteriocytes, sheath cells, and hemolymph [14], [29], [30]. FISH shows that those symbionts can be localized in secondary bacteriocytes and sheath cells (together making up part of the bacteriocytes) of one individual [31]. Artificial transfer of the symbionts by hemolymph injection [32] and microscopy observation [33] indicate the presence of the symbionts in the hemolymph. Those symbionts have major effects on the pea aphid's biology: *Hamiltonella* provides resistance to parasitoid wasps [34], *Serratia* provides resistance to high temperatures [35], [36] and some resistance to endoparasitoid wasps [34], *Regiella* enhances the aphid's ability to utilize specific host plants [37] and confers resistance to fungal infection [38], and *Rickettsia* negatively affects the aphid host's fitness [15]. However, it is not clear whether the same host can harbor the symbionts in the bacteriocytes and hemolymph simultaneously, and it would therefore be very interesting to check if the same effects on the host exist for the two different symbiont localizations (hemolymph and bacteriocytes).

Taken together, the results show that symbiont localization within its host is very diverse and likely stems from the nature of the host-symbiont relationship. However, the same symbiont may behave differently in different environments (e.g. inside and outside the bacteriocytes), and this phenomenon suggests a major effect of the symbiont's environment on its biology.
